# The Abuse of Quinine

**Published:** 1900-09

**Authors:** W. F. Church

**Affiliations:** Chicago, Ill., 1125 W. North Avenue


### THE ABUSE OF QUININE.

By W. F. CHURCH, M. D., Chicago, Ill.

THE history of therapeutics is full of illustrations, showing that every drug found valuable in a single condition has been tested repeatedly in almost every ailment known to man. This is due, in a measure, to the desire and search for specifics, but largely to the exaggerated notions commonly attached to a favorite remedy. Such has been the history especially of cinchona, or its chief alkaloid quinine. Originally used and introduced into Europe for malaria, its great efficacy in that disease naturally resulted in its extended trial and use for all symptoms and diseases. The difficulty in obtaining the drug and its high cost no doubt added to the estimate of its virtues and helped to create a great demand.

Medicine, like law, though not to the same extent, has respect for precedent and a drug may be used repeatedly without efficacious results and still its use be continued for no other reason than that it was recommended by someone in the past and that recommendation passed from text-book to text-book. Some of our latest authors on pharmacology do not base their work chiefly on original investigation, but compile their material from authors past and present. It need not, then, be a matter of wonder that all the advances in the art of therapeutics have failed to relegate quinine to its specific indications. It comes nearer being used as the universal remedy than any other.

There is no question but that the less the laity know and hear about morphine, acetanalid, sulphonal and kindred drugs, the better for them and the better for the profession. There are certainly excellent reasons for a common knowledge of the use of quinine in malarial infection that does not apply to other remedies for other conditions. Its value to settlers in a new country or travelers and explorers far from medical aid is hardly apt to be over estimated. But a chill is a chill to most people, whether it be from the plasmodium of Laveran, from an infection by pneumococci, or from whatever cause, and quinine is called into requisition, especially if they are living in what has been a malarial district. Thus, often valuable time is lost before a medical adviser is summoned.

That quinine is used so largely and universally is due chiefly to physicians themselves. If a learned doctor, in good repute, enthusiastically declares he broke up the grippe in his own case in twenty-four hours by two doses of quinine of 20 or 30 grains each taken in *spiritus fermenti*, is it any wonder his admirers, having such an example, attempt to accomplish a similar result with the same agents? Naturally it follows that quinine is often taken for influenza that is not influenza.

What difference does it make to the profession whether the laity take quinine or drench their internal organs with patent medicine? Very little probably if the ailment is slight, but a physician will hardly raise his standing with a patient who has been taking quinine without gratifying results if he finds it necessary to continue the drug. Not infrequently such a patient draws the conclusion that had he continued the use of the remedy his health would have been speedily restored; that under the circumstances the doctor did not deserve much credit and really displayed but little more skill than himself. A few may feel flattered because they are on the right track and think the doctor a good fellow in being able to recognise their wisdom. On the other hand, there are many willing to take quinine only when prescribed by themselves. A doctor should be sufficiently well versed in medical lore to effect a cure by other agents. The dictum that went forth from some great teacher in the past, "when you don't know what to give, give quinine," should not apply to this period. It should be inscribed only in the history of the time when men wrought in the morning twilight of our present science and fought disease with quinine as their greatest therapeutic weapon. Whoever goes forth now to fight the countless host of microörganisms, intending when in doubt to give the one remedy, cannot expect to become a good therapeutist or an expert diagnostician. A remedy, good in every case, can only beget carelessness in diagnosis.

Does the physiological and therapeutic action of quinine warrant its being repeatedly prescribed in every diseased condition from "ague to zoster?" The knowledge of the action of a drug so old and so widely used ought to be exact. However, works on therapeutics are not unanimous in their statements regarding its action on the blood, the most important of all. There is no doubt that it acts as an efficient poison to the malarial plasmodium, but it is highly important that we should have an exact knowledge of its action on the corpuscles in other infections or in anemia. Are the red corpuscles increased or decreased? Is their oxygen-carrying power increased or diminished? Is there any change in the number of white cells? Hare says: "Quinine, in medicinal amounts, increases the number of red-blood corpuscles very materially." Foster's Therapeutics states that "the red cells are slightly diminished in number, (a statement directly opposite to the first authority), the coagulability of the blood is diminished and emigration of cells embarrassed; also there is diminution of oxygen-carrying properties." Some experimenters and authors, among them Potter, declare that leucocytosis follows its use while others maintain that the white cells are diminished in number.

In order to reach a correct conclusion from these contradictory statements, it will be necessary to decide from the weight of testimony, giving due consideration to careful investigation. From the dozen authors consulted, I shall only take space to give the views of two of the latest, who present, according to my judgment, the most reliable results. These do not materially differ from those given by Foster, but are more positive:

Cushny (1899) says:

The number of leucocytes in the blood is much diminished by the administration of quinine in man and the lower animals, but it is unknown how this is effected. The statement that the normal spleen undergoes a contraction in size and partial atrophy after quinine, while not improbable in itself, is not supported by experiments in which accurate methods were used. The change in the circulation in mammals are caused by a preliminary contraction of the arterioles and acceleration of the heart, followed by dilatation of the former and slowing and weakening of the latter.

Schleif and Gallaudet (1899) give a brief summary as follows:

Ameboid activity of the white corpuscles is decidedly lessened and their number is diminished; the red corpuscles are decreased in size and the power of their hemoglobin to carry oxygen and ozone is impaired.

From this evidence the deduction is legitimately made that quinine is not the proper remedy when leucocytosis is desired

According to Nancrede (*Prin. of Surg.*, 1899):

The sole exciting causes of inflammation are pathogenic organisms or their products. Pus consists of pus cells, *i.e.*, dead and dying leucocytes and germinal cells, which have perished in their attack upon the germs or been killed by the germ products.

Knowing, then, the action of the white cells on microörganisms, it seems that the proper mode of treatment in some types of inflammation would be to increase the number of leucocytes, if they were being overcome.

The authority quoted above (Schleif and Gallaudet) further says:

In conditions of septic intoxication, pyemia and puerperal fever and anemia, it has been much in vogue, but evidence of its utility is wanting.

The tonic properties rest largely on the belief in the increase of the red corpuscles, but here there is a difference of opinion and the negative side seems to have the greatest weight of authority. This side also maintains that the oxygen-carrying power is diminished. It may be that the tonic effects are accomplished in some cases by its ability to increase the appetite, but many stomachs revolt at its use and there are undoubtedly superior stomachics. Increased action of the heart in ordinary cases is probably also an important factor, though temporary. As an antipyretic, with the single exception before mentioned, quinine now occupies an inferior position though Jacobi claims it tends to prevent the destruction of cells, and is consequently valuable. It is thought by others that a lessened amount of nitrogenous tissue is destroyed. In select cases of impaired matabolism, when the nitrogenous waste is excessive, it would be of value. An opposite condition might be a contraindication. However valuable quinine may be in aborting coryza if used early, as claimed by some, it can but aggravate the unpleasant feeling of fulness when symptoms are fully developed. As a local application its use would certainly be preferable to internal administration.

To afford a proper basis for comparison it may be well to state here the legitimate uses, as given in one of the latest books (Cushny). Quinine is there recommended in malaria, in some cases of leucemia enlargement, in febrile conditions (though inferior except when temperature is falling) and in various forms of neuralgia and headache, especially when periodic in appearance. How many practitioners limit the use of quinine to the legitimate indications? If it is necessary to give a placebo, why not give something that is a genuine placebo and not a drug capable of producing in some instances unpleasant or even harmful results? Occasionally a patient is peculiarly susceptible. Foster's Therapeutics states: "The toxic dose is difficult to estimate. Forty-four grains in divided doses in fifty hours, in one instance, produced death and twelve grains are recorded as having caused amaurosis." It is well-known that large doses may cause complete blindness or deafness, which, as a rule, gradually disappears, but may in rare instances remain permanent.

A few years since a physician in Toledo lost prestige in a family, and, of course, among the circle of friends, by producing amaurosis with large doses of quinine. A case of quinine blindness was recently reported by Dr. Moulton in the *Western Clinical Recorder*, in a child of three years. In the course of five days 90 grains were administered. On the sixth day the child was blind. After the discovery 20 grains more were given in the course of two days. The treatment consisted of Fowler's solution and strychnine. The child could see a little twelve weeks after the accident, and in six weeks more could pick up small objects, "as pencils and even pins from the floor." In Moulton's opinion the accident of quinine blindness is not so rare as generally supposed, especially partial and transient impairment of vision. Susceptibility may be manifested in other ways. My attention was recently called to the case of a child in whom quinine always produced an erythema, in one instance, accompanied by grave symptoms.

At a recent meeting of the Pittsburg Medical Society (*Phil. Med. Jour.*):

Dr. J. D. Heard reported a case of extreme susceptibility in a young girl. One-eighth of a grain sufficed to cause an acute universal erythema with desquamation and headache. Dr. F. F. Simpson reported two similar cases, one suffering general desquamation, including nails, the other going into collapse and recovering under stimulation. Dr. P. J. Eaton reported a case in which quinine produced intense prolonged headache and insomnia. It is fair to presume that similar results are produced more frequently than is supposed as the cases may not be recognised, or if so, not reported. If other medical societies could report a proportional number of cases, and there is no reason to think that Pittsburg has a monopoly in this line, the list might open the eyes of the firm believer in quinine's harmlessness. There are excellent reasons to suppose that few of the unpleasant results are reported, especially in the country and smaller cities. Cases might be recognised by opponents and the opportunity taken to discredit the physician reporting.

On page 319 of the *Canadian Journal of Medicine and Surgery*, Dr. A. J. Harrington says:

> The craze for ingesting quinine in every possible disease has caused an enormous amount of nervous disorder among business men, who keep the drug in their pockets just as they do car tickets.
>
> Many of the most serious cases which come under my notice are due to this drug. I feel safe in asserting that quinine causes more trouble to the community than morphine, although we do not hear so much about it. Drinkers and topers of opium and cocaine are spotted by most of their acquaintances, but the nerve tremor due to the salts of cinchona are put down to overwork, etc. Even medical men are frequently deceived by the symptoms, for I have had many cases sent to me where the cause was never suspected by the family doctor.

Koch, Bastianelli and other close observers, claims that hemoglobinuria may be produced in some cases when given for malaria and also when used for prophylaxis. Giving quinine for conditions carelessly diagnosticated as malaria, but in reality not, is without doubt its greatest abuse. Osler recently said: "Malaria is such an accommodating word. It covers such a multitude of diagnostic sins. It was at least as consoling in its way as the unctious word, Mesopotamia, to the old woman in the story." He who does not believe that every diseased condition, not easily diagnosticated, should be called malaria and has practised where some physicians had that habit and treated accordingly, will appreciate his arraignment of the distorted application of the term. During the first years of my practice, it was hard to understand why it was impossible for me to discover malaria when so many cases were reported by others. Light finally dawned. Malaria was the much abused term for uncertainty in diagnosis. Dr. O'Brien, of New York, recently in a medical meeting called attention to the frequency with which patients were given quinine for a supposed malaria when the tonsils were the real seat of the infection. The plasmodium has received too much credit for effects wrought by the typhoid bacillus. Altogether too frequently for the welfare of the patient have the chills and fever of pus formation been mistaken for intermittent fever and treated accordingly. A proper use of the microscope in diagnosis will give a more positive knowledge to the physician, serve to increase his prestige and assure the patient the benefit of the proper drugs.

Quinine is contraindicated in inflammation of the middle-ear and labyrinth, in renal irritation, and in acute inflammation of the gastro-intestinal tract. Without inflammation, in some instances, it is not well borne and may produce a heaviness, depression, or even vomiting, and more or less irritation of the digestive canal. Not a small number of individuals have learned of these unpleasant effects by dosing themselves and beseech their medical attendant not to give them quinine. Although quinine is not used now by physicians to the same extent as formerly, undoubtedly the amount is much too large. An agent for a physicians' supply house told me recently that his sales averaged about twenty-five ounces a day and he generally called on five or six physicians. His visits were two months apart.

According to official reports the people of the United States consume one-third of the quinine used in the world. In 1898, 1,539,056,750 grains were imported, or 20 grains to every man, woman and child. The excess used is certainly an abuse of an excellent drug and for the reasons above enumerated. Learned professors are partly responsible, as they advise it so frequently that students learn to recommend it "on general principles" in almost every case. Routinists, even after years of practice, are not excusable on the ground that what was good enough for the parents will do for the children. Fortunately the torch of science does not light the way for those deep in the rut and contented therein, but only for those who toiling on, lay aside habit and prejudice and accept the truth.

1125 W. NORTH AVENUE.

